# HIV and Bone Disease: A Perspective of the Role of microRNAs in Bone Biology upon HIV Infection

**DOI:** 10.1155/2013/571418

**Published:** 2013-10-27

**Authors:** Fabiola E. Del Carpio-Cano, Raul A. DeLa Cadena, Bassel E. Sawaya

**Affiliations:** Department of Physiology, Fels Institute for Cancer Research and Molecular Biology, Temple University School of Medicine, 3307 North Broad Street, Philadelphia, PA 19140, USA

## Abstract

Increased life expectancy and the need for long-term antiretroviral therapy have brought new challenges to the clinical management of HIV-infected individuals. The prevalence of osteoporosis and fractures is increased in HIV-infected patients; thus optimal strategies for risk management and treatment in this group of patients need to be defined. Prevention of bone loss is an important component of HIV care as the HIV population grows older. Understanding the mechanisms by which HIV infection affects bone biology leading to osteoporosis is crucial to delineate potential adjuvant treatments. This review focuses on HIV-induced osteoporosis within the context of microRNAs (miRNAs) by reviewing first basic concepts of bone biology as well as current knowledge of the role of miRNAs in bone development. Evidence that HIV-associated osteoporosis is in part independent of therapies employed to treat HIV (HAART) is supported by cross-sectional and longitudinal studies and is the focus of this review.

## 1. Introduction

The emergence of human immunodeficiency virus (HIV) disease was initially reported in 1981 followed by the identification of the HIV as the cause of the disease in 1983. HIV is a global pandemic that has become the leading infectious disease killer of adults worldwide. In 2006, more than 65 million people had been infected with HIV worldwide and 25 million had died of HIV. In 2007, it was estimated that 33 million people were living with HIV with 2.7 million new infections and 2 million deaths each year. This has caused tremendous socioeconomic damage worldwide. In the USA, this condition afflicts the African American population in a disproportionate fashion although all racial and ethnic groups are afflicted [1]. There are new modalities of treatment including bone marrow transplantation [2], the “Berlin Patient” receiving transplantation with cells conferring HIV resistance [3], and members of the Visconti cohort who seem to be free of HIV infection after stopping antiretroviral therapy [4]. However, all of the above-mentioned modalities of treatment are raising complex ethical issues [5], and it will take years before being fully implemented if approved.

Among individuals living with HIV, the proportion of deaths attributed to chronic noninfectious comorbid diseases has increased over the past 15 years. This is partly a result of increased longevity in the area of highly active antiretroviral therapy (HAART) and also because HIV infection is related, causally or otherwise, to several chronic conditions. These comorbidities include conditions that are strongly associated with modifiable risk factors, such as diabetes and cardiovascular, renal, and bone diseases [6].

This review focuses on HIV-induced osteoporosis within the context of microRNAs (miRNAs) by reviewing first basic concepts of bone biology as well as current knowledge of the role of miRNAs in bone formation. The literature on miRNAs and HIV and bone is scarce when compared to miRNAs and bone biology. Evidence that HIV-associated osteoporosis is in part independent of therapies employed to treat HIV (HAART) is supported by cross-sectional and longitudinal studies and elegantly recently reviewed [7]. A recent publication strongly supports such evidence, namely, a population-based cohort study that revealed a strong association between HIV infection and hip fracture incidence, with an almost fivefold increased risk in the HIV patients which was found to be independent of sex, age, smoking, alcohol drinking, and comorbidities [8]. The effect of HAART in bone biology is beyond the scope of this review.

## 2. Bone Biology

Bone is formed through two distinct phases: endochondral ossification, where cartilage is replaced by bone, and intramembranous ossification where bones are shaped directly from condensation of mesenchymal cells without intermediate cartilage. Bone is continuously remodeled throughout life, and an imbalance in this process can result in bone disease.

The balance between osteoblast bone matrix deposition and osteoclast-mediated bone resorption is an essential feature of homeostatic bone remodeling. This process allows the regulation of skeletal growth and repair and the maintenance of skeletal integrity. Osteoblasts and osteoclasts are derived from different cell lineages, with osteoblasts being formed by the differentiation of mesenchymal stem cells (MSCs) precursors and osteoclasts derived from hematopoietic stem cells.

Mesenchymal stem cells are pluripotent cells that arise from different tissues sources. Bone marrow stromal cells are able to differentiate into several mesenchymal cell types, including osteoblasts, myocytes, chondrocytes, neurons, and adipocytes [9]. Osteogenic differentiation of MSCs is driven by intercellular signaling systems that include bone morphogenic proteins (BMPs), Wnt ligands, transforming growth factor-*β* (TGF-*β*), fibroblast growth factor (FGF), hedgehog signaling pathways, hormones, cytokines, and other growth factors [10–12]. In addition, this process is also controlled at the genetic and epigenetic level including transcriptional regulation by DNA methylation, nucleosomal modifications, and protein modifications [13]. MSCs differentiation is a tightly regulated process that remains under investigation. Incomplete understanding of MSCs differentiation represents a potential challenge in the understanding of HIV-associated osteoporosis.

TGF-*β*/BMPs have been widely recognized as playing an intricate role in bone formation during mammalian development [10, 12–14]. Their signaling transduction is through both canonical Smad-dependent pathways (TGF-*β*/BMP ligands, receptors, and Smads) and noncanonical Smad-independent signaling pathways (e.g., p38 mitogen-activated protein kinase pathway, MAPK). Following TGF-*β*/BMP induction, both the Smad and p38 MAPK pathways converge at the Runt-related transcription factor 2 (Runx2) gene to control mesenchymal precursor cell differentiation [11].

Runx2, also known as Cbf*α*1, Osf2, and AML3, is considered as the first master transcription factor responsible for the acquisition of osteo-chondrogenic characteristics and for the concomitant repression differentiation towards the adipocytic phenotype [12]. Downstream of Runx2 the fate of the bone precursor is mainly determined by the expression of Osterix (Sp7). Osterix belongs to the Sp subfamily of Sp/XKLF transcription factors. Osterix acts in a complex with the nuclear factor of activated T-cells-C1 (NFATc1) and activates the transcription of bone-related genes, including collagen type 1 (COL1A1) and osteocalcin (BGLAP) [15]. The importance of this bone-specific transcription factor in osteoblast differentiation and bone formation has been demonstrated in Osterix null-mice, where, while the ability to form cartilage was retained, bone formation was impaired due to the complete absence of osteoblasts [15].

Except for BMP-1, all BMPs belong to the transforming growth factor-*β* (TGF*β*) superfamily [16–19]. BMPs enhance the expression of alkaline phosphatase (ALP), parathyroid hormone (PTH), PTH-related protein receptor (PTHrP), type I collagen and osteocalcin by signaling through other TGF-*β* superfamily members, which are serine/threonine kinase receptors [10]. Smad transcription factors are substrates for the receptor kinases and thus involved in osteoblast differentiation. Among the BMP2-induced genes important for osteoblast development is the zinc finger transcription factor, Osterix described above. Further upstream in this pathway is the cell cycle checkpoint protein and tumor suppressor p53 [20]. Deficiency in p53 leads to osteoclastogenesis [20] in P53^−/−^ mice. Noggin, also known as NOG, prevents BMP2 receptor binding, providing a tool to interrupt the BMP-p53-Cbfa1-Osterix axis in the osteoblast lineage [21]. In addition to Runx2, PPAR*γ* (peroxisome proliferators activator receptor), another transcription factor, drives a proadipogenic phenotype [22]. Alterations in the ratio of osteoblasts to adipocytes generated from MSCs have been clinically linked to decreased bone mass, with an increase in bone marrow adipocyte content observed in both osteoporosis and osteopenia [22, 23]. Other conditions which lead to bone loss, such as treatment with glucocorticoids [22, 24], have also been shown to increase the number of bone marrow adipocytes.

Wnt/*β*-catenin [25] promotes new bone formation by functioning as a positive regulator of osteoblasts [26–28] and by upregulating osteoprotegerin (OPG) [29–31] and downregulating the receptor activator of nuclear factor *κ*_*B*_ ligand (RANKL) [30, 32], which are both important in osteoclastogenesis. Wnt/*β*-catenin signaling in the osteoblast lineage is activated by binding of canonical Wnt ligands, such as Wnt3a, to a membrane-bound receptor complex that consists of Frizzled and low-density-lipoprotein receptor-related protein 5/6 (LRP5/6). Canonical Wnt ligands inhibit the degradation of *β*-catenin in the cell nucleus where it cooperates with transcription factors of the T-cell factor/lymphoid enhancer factor family in regulating target gene expression [33, 34]. In humans subjects diminished Wnt/*β*-catenin signaling because loss of function in LRP5 leads to osteoporosis [35].

Development of osteoclasts proceeds within the local microenvironment of bone. Several lines of evidence indicate that osteoclast progenitors are hematopoietic cells of the monocyte/macrophage lineage. Macrophage colony-stimulating factor (M-CSF) [36] also known as colony stimulating factor-1 (CSF-1) is produced by osteoblasts/stromal cells and plays an essential role in osteoclast development. The discovery of the tumor necrosis factor-ligand family has clarified the precise mechanism by which osteoblasts/stromal cells regulate osteoclast differentiation and function. OPG [37] is a secreted protein expressed by a wide range of cells including those in the heart, kidney, lung, and especially osteoblastic cells. OPG is a potent negative regulator of osteoclastogenesis. Its ligand was identified and originally named osteoprotegerin ligand (OPGL). This ligand was later identified as RANKL, a member of the TNF superfamily of ligands. Unlike OPG, RANKL is a potent positive regulator of osteoclastogenesis by binding to its own receptor RANK [38]. The discovery of the OPG-RANK-RANKL axis defined the hallmark mechanism of osteoclastogenesis [39]. RANKL is expressed in osteoblasts and forces the cells to physically interact with osteoclastic precursors in order for RANKL to bind to its receptor RANK. To negatively regulate this mechanism, OPG is expressed by osteoblasts and acts as a decoy receptor to compete with RANKL for RANK [40]. Thus, this axis allows osteoblasts to directly regulate osteoclasts both positively and negatively. Fas-ligand (FASGL or CD95L) is a type-II transmembrane protein that belongs to the tumor necrosis factor (TNF) family. Its binding with its receptor induces apoptosis, and there is evidence as described below to play a role on osteoclasts mediated by the action of estrogens [41].

## 3. Pathophysiology of Osteoporosis

Osteoporosis is a systemic skeletal disease characterized by low bone mass and microarchitectural deterioration of bone tissue, with a consequent increase in bone fragility and susceptibility to fracture. The disease is characterized by an imbalance of bone remodeling favoring osteoclastic bone resorption. Bone strength is affected by two main factors, namely, bone density and bone quality. Using dual energy X-ray absorptiometry (DEXA), a patient can be evaluated for bone mineral density (BMD).

Today the literature of miRNAs in osteoporosis is scarce but becoming available. There are examples of both inhibition of osteblastogenesis as well as osteoclastogenesis. For instance, Wnt inhibitors targeted by miRNAs (miRNA 218) have been described in the literature [42] as well as inhibition of the action of specific miRNAs (miRNA-148a) from blocking key players like transcriptor factor V-maf musculoaponeurotic fibrosarcoma oncogene homolog B (MAFB) and thus promoting a proosteoclastic profile [43]. Osteoporosis is commonly associated with postmenopausal women and to a lesser extent elderly men. The induction of miR-21 during osteoclastogenesis is antagonized by estrogen [41]. Estrogen normally reduces miR-21 expression in osteoclasts, and this reduction depresses the expression of miR-21 target Fas-ligand [41] (FASLG). This finding suggests that miR-21 may play a role in reducing bone resorption in premenopausal women with robust estrogen levels [34]. The high incidence of osteoporosis in postmenopausal women is due to the change in hormonal balance associated with menopause. Estrogen, a potent endocrine hormone linked to osteoporosis, has been demonstrated to bind osteoblastic cells and suppress the expression of several paracrine proosteoblast factors including interleukin-1 (IL-1), IL-6, and tumor necrosis factor-*α* (TNF*α*) [44]. Furthermore, estrogen can increase OPG and inhibit RANKL expression, thus explaining the osteoporosis seen in menopause [45–48]. Estrogen has been shown to inhibit the transcription factor Egr-1 (early growth response protein-1) which in turn increases the production of M-CSF and thus bone resorption [49, 50]. All of these molecules are expressed by osteoblasts which can bind to osteoclasts suggesting that estrogens can indirectly affect intracellular communication between osteoclasts and osteoblasts, but more investigation is required for this last statement.

## 4. Biology and Function of miRNA

miRNAs have been involved in almost every biological process, including development timing, cell differentiation, cell proliferation, cell death, metabolic control, transposon silencing, and antiviral defense [51]. In humans, a significant number of miRNAs have been identified which are evolutionarily conserved [52]. About 40–90% of the human protein encoding genes are under miRNA-mediated gene regulation [53]. miRNA genes are found as single or clustered transcription units and expressed from the intron regions of protein-coding or non-protein-coding genes [54, 55]. Some are expressed as independent transcription units. The steps involved in miRNA synthesis include the generation of a primary miRNA in the nucleus. The miRNA is then processed into a stem-loop-structured pre-miRNAs. The pre-miRNA is then transported into the cytoplasm and cleaved. At this point, the double-stranded miRNA is transient and subsequently incorporated into a multicomponent protein called the RNA-induced silencing complex (RISC) containing the Argonaute 2 protein. In the end, one strand of the miRNA is selected as a mature miRNA while the second one is degraded. Mature miRNAs negatively regulate gene expression via translational repression. In mammalian cells, most miRNAs have imperfect ability to target mRNAs resulting in reduced expression of the corresponding proteins.

## 5. MicroRNA Regulatory Role in Bone Biology

The onset of osteoporosis has been attributed to decreased osteoblast function and/or increased osteoclast activity. A significant number of miRNAs [56–62] have been reported to be involved in osteoblast differentiation and bone formation. For instance, miRNA-2861 promotes osteogenesis by targeting the genes encoding for histone deacetylase 5 (HDAC5) [63] and transcriptor factor homeobox protein (Hoxa2) [64]. HDAC5 is an enzyme, which plays a critical role in transcriptional regulation, cell cycle progression, and developmental events. Histone acetylation/deacetylation alters chromosomal structure and affects transcription factor access to DNA. Hoxa2 is a protein and a member of a class of transcription factors called homeobox genes and found in clusters named A, B, C, and D on four separate chromosomes. Expression of these proteins is spatially and temporally regulated during embryonic development. Contributing to bone formation, inducible brown adipogenesis is mediated by miRNA-196a by suppressing the expression of the white-fat gene Hoxc8 [7, 65]. Osteoblastic differentiation is downregulated by miRNA-125b by targeting osteocalcin [57] and human epidermal growth factor receptor-2 (ERB*β*2) [57]. Canonical Wnt signaling, which is increased during osteoblastic differentiation, induces the expression of miRNA-29a with subsequent suppression of osteonectin [61]. Signaling of this last pathway is stimulated in mature osteoblasts, which is anabolic for bone by targeting Dickkopf-related protein 1 (DKK1) by miRNA-335 [66, 67]. DKK1 is a negative regulator of Wnt signaling. Inhibition of osteoprogenitors has been shown to be linked to attenuation of Runx2 and Smad5 by miRNA-133 and miRNA-135, respectively [59, 68]. Alternatively promotion of osteogenesis can be induced by downregulation of known inhibitors of osteoblast differentiation, namely, HDAC4, TGF*β*3, activin receptor type 2A (ACVR2A), beta-catenin-interaction protein 1 (CTNNBIP1), and dual specificity protein phosphatase 2 (DUSP2) by miRNA-29b [69]. ACVR2A is a protein and receptor and belongs to the TGF*β* superfamily of structurally related signaling pathways. CTNNBIP1 prevents the interaction with T-cell transcriptor factor (TCF) and thus a negative regulator of Wnt signaling. DUSP2 is an enzyme that inactivates phosphorylation of serines and threonines on its targets and thus involved in MAPK/ERK, SAPK/JNK, and p38 signaling pathways. PTK2 and osterix, which are proteins highly expressed in human MSC, are the target of miRNA-138. miRNA-138 inhibits the osteoblast focal adhesion kinase pathway and osterix, which is required for osteoblast differentiation [70]. BMP/activin membrane-bound inhibitor homolog (BAMBI) is a protein related to the type-I receptors of the TGF*β* family and in conjunction with cysteine-rich motor neuron-1 protein (CRIM1) are targets of miRNA-20a, increasing BMP signaling by targeting antagonists of the pathway, in addition to targeting PPAR*γ* which represses adipogenesis [71]. Activating transcription factor 4 (ATF4), a gene encoding one of the main proteins required for osteoblast function, is a target for miRNA-214. Osterix is the target of miRNA-637, which promotes adipogenesis and inhibits osteoblast differentiation via osterix downregulation [72]. Thus, as the biology of osteoblastogenesis by growth factors and inhibitors is complex in nature, its regulation by miRNAs is equally intricate and provides valuable information regarding pathways potentially affected during early and late HIV infection. Noteworthy to mention is that an effect on osteoblastogenesis translates into a potential effect in osteoclastogenesis due to the nature of osteoblast bone biology.

Three microRNAs, namely, miR-96, miR-124, and miR-199a, are differentially expressed during mesenchymal lineage commitment in mouse MSCs [73, 74]. In humans miR-138 was found downregulated during osteogenesis [70]. In cell culture models microRNA expression during osteoblastic differentiation has been documented [59, 69, 75]. The overall outcome from such studies is that BMP2 controls the switch between muscle and bone formation by regulating miRNA expression [59]. Among the miRNAs involved are miR-133 and miR-135, both collectively suppressing RUNX2 and SMAD5. This last effect of BMP2 allows the flow of the BMP2-RUNX2-SMAD axis toward osteoblast differentiation [59]. When cell cultures of precommitted cells are used instead of during lineage commitment, the story is a bit different, with the identification of miR-29 and miR-26 involved in the production of extracellular matrix collagens type I, IV, and V. Moreover, miR-29 seems to be involved in osteoblast maturation by regulating the TGF*β* pathway and Wnt and MAPK signaling and by downregulation of HDAC4 [63]. This last observation, namely, an effect on TGF*β*, Wnt, and MAPK pathways by miRNAs, has been recently documented in humans and discussed further under last section [76].Transcriptional suppression of the miR cluster 23a-27a-24-2 by RUNX2 promotes osteoblastogenesis. Another pathway identified as promoting osteoblast differentiation is the enhancement of Wnt signaling via miR-218 [42].

The best studied miRNAs that are involved in differentiation of osteoclasts are miR-21, miR-155, and miR-223 [41, 75, 77–79]. Expression of miRNA-21 was reported to be controlled by the transcription factor c-Fos [75], where RANKL-induced c-Fos upregulation of miRNA-21 downregulates programmed cell death protein 4 (PDCD4) expression levels. In a cell line RAW264.7 an osteoclast precursor, miRNA-223 was found to play a role in osteoclast differentiation. PU.1, a transcription factor induced by M-CSF, stimulates the expression of miRNA-223, which in turn downregulates the NFI-A levels required for upregulating macrophage colony-stimulating factor receptor (M-CSFR) [7, 65, 78, 79]. PU.1 selectively regulates genes during osteoclast differentiation. The result of this is the increased expression of transcription factors PU.1, MITF, and c-Fos. The increases are induced by M-CSF and RANKL through upregulated M-CSFR and RANK with the consequent differentiation of cells into activated osteoclasts. The paradoxical results of miR-223 being both supportive and inhibitory for osteoclastogenesis are reminiscent of the opposing results for miR-2018 which enhances osteoblastogenesis while attenuating the bone-specific activity of RUNX2. In both cases, negative feedback circuits may be triggered to prevent precocious differentiation or to avoid hypercatalytic differentiation of bone cells, which in each case would perturb normal bone deposition and remodeling [34]. In human circulating monocytes, miRNA-133a has been identified as a biomarker associated with postmenopausal osteoporosis [80]. Despite the role of estrogens in this particular study, miRNA-133a should be further investigated. The V-maf musculoaponeurotic fibrosarcoma oncogene homolog B (MAFB) transcription factor, which negatively regulates RANKL-induced osteoclastogenesis, is regulated by miRNA-148a [43]. Therefore, the biology of osteoclastogenesis is just as complex as that for osteoblastogenesis, is in part dependent on the biology of osteoblasts, and thus, not surprisingly, highly regulated by specific miRNAs which may be affected during HIV infection.

## 6. HIV and Bone Biology

The reported prevalence of osteopenia in HIV-infected cohorts has ranged from 22% to 71% with rates of osteoporosis varying from 3% to 33% [81–87]. The pathogenesis of osteoporosis in HIV infection is multifactorial including traditional factors such as smoking and alcohol. However, as mentioned in the introduction, a recent study has elegantly ruled out comorbidities and the influence of smoking and alcohol [8]. Thus, the viral infection itself is involved in the pathogenesis. Indeed another recent publication analyzed the expression level of 754 miRNAs of 10 HIV seropositive and 10 seronegative individuals. Algorithms were employed to predict potential target genes and pathway analysis. A total of 56 significantly differentially expressed miRNAs were identified by microarray. Among them, 49 miRNAs were downregulated and 7 were upregulated in the seropositive patients. Predicted target genes were mainly involved with MAPK, TGF*β*, and Wnt pathways [76]. Therefore, the work in the area of the role of miRNAs in osteoblastogenesis and osteoclastogenesis is starting to provide some fruit in view of the findings in the study cited above [76].

Proinflammatory cytokines released during HIV infection include TNF*α* [88] and OPG which affect osteoblast and osteoclast development [89, 90]. Studies support the notion of HIV proteins favoring osteoclast activation [91] by affecting the axis comprised of OPG-RANK-RANKL [91, 92]. HIV viral protein R (Vpr) and HIV envelope glycoprotein, gp120 can both upregulate RANKL [93, 94]. HIV infection upregulates TNF-related apoptosis-inducing ligand (TRAIL) which in turn binds to OPG, limiting the capacity of OPG to regulate RANKL-induced osteoclast activation [95, 96].

The following events are noteworthy to mention because of their association with osteoclastogenesis. For instance, osteoclastogenesis is facilitated by RANK signaling of tumor necrosis factor-receptor associated factor 6 (TRAF-6) phosphorylation of JNK and Akt [48, 97]. Activation of extracellular-signal-regulated kinases (ERK) signaling by RANKL has been documented and mediated by gp120 [93]. Increased osteoclast viability has been postulated to occur due to a lack of response to oxidative stress in mitochondria, [98] preventing apoptotic cell death in HIV. TNF*α* has also been shown to induce apoptosis of osteoblasts as a result of HIV infection, potentiating bone loss [88].

## 7. Summary

During bone formation, subsets of miRNAs function at both early and late stages of osteoblast and osteoclast differentiation to regulate the expression of various growth factors and inhibitors of pathways operative in these cells. How numerous miRNAs involved in the control of bone formation and remodeling coordinate the activities of these pathways is an area of investigation. A current hypothesis postulates that all types of RNA transcripts comprise large-scale regulatory networks [99].

HIV infection results in a multitude of changes in infected cells. During periods of active viral replication, these changes occur in multiple classes of molecules, including mRNAs and proteins [100, 101]. Small RNAs constitute an additional class of molecules whose expression is likely to be altered by HIV infection. Among the best-characterized types of small RNAs are microRNAs, single-stranded RNA molecules discussed above. HIV infection has been shown to alter microRNAs expression both in cultured cells and in peripheral blood mononuclear cells [102, 103]. Next-generation sequencing of small RNAs from HIV-infected cells has identified phased microRNA expression patterns and candidate novel microRNAs differentially expressed upon infection [104] for future investigation.

## References

[1] Division of HIV/AIDS Prevention, CDC, National Center for HIV/AIDS VH, STD and TB Prevention (2012). *HIV in the United States: At a Glance*.

[2] McNeil DG (July 2013). After marrow transplants, 2 more patients appear H.I.V.-free without drugs. *New York Times*.

[3] Hutter G, Nowak D, Mossner M (2009). Long-term control of HIV by CCR5 delta32/delta32 stem-cell transplantation. *The New England Journal of Medicine*.

[4] Saez-Cirion A, Bacchus C, Hocqueloux L (2013). Post-treatment HIV-1 controllers with a long-term virological remission after the interruption of early initiated antiretroviral therapy ANRS VISCONTI Study. *PLoS Pathogens*.

[5] Sugarman J (2013). HIV cure research: expanding the ethical considerations. *Annals of Internal Medicine*.

[6] Peters B, Post F, Wierzbicki A (2013). Screening for chronic comorbid diseases in people with HIV: the need for a strategic approach. *HIV Medicine*.

[7] Stone B, Dockrell D, Bowman C, McCloskey E (2010). HIV and bone disease. *Archives of Biochemistry and Biophysics*.

[8] Guerri-Fernandez R, Vestergaard P, Carbonell C (2013). HIV infection is strongly associated with hip fracture risk, independently of age, gender, and comorbidities: a population-based cohort study. *Journal of Bone and Mineral Research*.

[9] Caplan AI (1991). Mesenchymal stem cells. *Journal of Orthopaedic Research*.

[10] Yamaguchi A, Komori T, Suda T (2000). Regulation of osteoblast differentiation mediated by bone morphogenetic proteins, hedgehogs, and Cbfa1. *Endocrine Reviews*.

[11] Li Y-L, Xiao Z-S (2006). Advances in Runx2 regulation and its isoforms. *Medical Hypotheses*.

[12] Tou L, Quibria N, Alexander JM (2003). Transcriptional regulation of the human Runx2/Cbfa1 gene promoter by bone morphogenetic protein-7. *Molecular and Cellular Endocrinology*.

[13] Jeon E-J, Lee K-Y, Choi N-S (2006). Bone morphogenetic protein-2 stimulates Runx2 acetylation. *Journal of Biological Chemistry*.

[14] Mbalaviele G, Sheikh S, Stains JP (2005). *β*-catenin and BMP-2 synergize to promote osteoblast differentiation and new bone formation. *Journal of Cellular Biochemistry*.

[15] Sinha KM, Zhou X (2013). Genetic and molecular control of osterix in skeletal formation. *Journal of Cellular Biochemistry*.

[16] Hogan BLM (1996). Bone morphogenetic proteins: multifunctional regulators of vertebrate development. *Genes and Development*.

[17] Massague J (2000). How cells read TGF-beta signals. *Nature Reviews Molecular Cell Biology*.

[18] Massagué J, Chen Y-G (2000). Controlling TGF-*β* signaling. *Genes and Development*.

[19] Miyazono K, ten Dijke P, Heldin C-H (2000). TGF-*β* signaling by Smad proteins. *Advances in Immunology*.

[20] Wang X, Kua H-Y, Hu Y (2006). p53 functions as a negative regulator of osteoblastogenesis, osteoblast-dependent osteoclastogenesis, and bone remodeling. *Journal of Cell Biology*.

[21] Nohe A, Keating E, Knaus P, Petersen NO (2004). Signal transduction of bone morphogenetic protein receptors. *Cellular Signalling*.

[22] Gregory CA, Prockop DJ, Spees JL (2005). Non-hematopoietic bone marrow stem cells: molecular control of expansion and differentiation. *Experimental Cell Research*.

[23] Nuttall ME, Gimble JM (2004). Controlling the balance between osteoblastogenesis and adipogenesis and the consequent therapeutic implications. *Current Opinion in Pharmacology*.

[24] Meunier P, Aaron J, Edouard C, Vignon G (1971). Osteoporosis and the replacement of cell populations of the marrow by adipose tissue. A quantitative study of 84 iliac bone biopsies. *Clinical Orthopaedics and Related Research*.

[25] Hartmann C (2006). A Wnt canon orchestrating osteoblastogenesis. *Trends in Cell Biology*.

[26] Hill TP, Später D, Taketo MM, Birchmeier W, Hartmann C (2005). Canonical Wnt/*β*-catenin signaling prevents osteoblasts from differentiating into chondrocytes. *Developmental Cell*.

[27] Day TF, Guo X, Garrett-Beal L, Yang Y (2005). Wnt/*β*-catenin signaling in mesenchymal progenitors controls osteoblast and chondrocyte differentiation during vertebrate skeletogenesis. *Developmental Cell*.

[28] Hu H, Hilton MJ, Tu X, Yu K, Ornitz DM, Long F (2005). Sequential roles of Hedgehog and Wnt signaling in osteoblast development. *Development*.

[29] Glass DA, Bialek P, Ahn JD (2005). Canonical Wnt signaling in differentiated osteoblasts controls osteoclast differentiation. *Developmental Cell*.

[30] Holmen SL, Zylstra CR, Mukherjee A (2005). Essential role of *β*-catenin in postnatal bone acquisition. *Journal of Biological Chemistry*.

[31] Jackson A, Vayssière B, Garcia T (2005). Gene array analysis of Wnt-regulated genes in C3H10T1/2 cells. *Bone*.

[32] Spencer GJ, Utting JC, Etheridge SL, Arnett TR, Genever PG (2006). Wnt signalling in osteoblasts regulates expression of the receptor activator of NF*κ*B ligand and inhibits osteoclastogenesis in vitro. *Journal of Cell Science*.

[33] Logan CY, Nusse R (2004). The Wnt signaling pathway in development and disease. *Annual Review of Cell and Developmental Biology*.

[34] van Wijnen AJ, van de Peppel J, van Leeuwen JP (2013). MicroRNA functions in osteogenesis and dysfunctions in osteoporosis. *Current Osteoporosis Reports*.

[35] Gong Y, Slee RB, Fukai N (2001). LDL receptor-related protein 5 (LRP5) affects bone accrual and eye development. *Cell*.

[36] Karsenty G, Wagner EF (2002). Reaching a genetic and molecular understanding of skeletal development. *Developmental Cell*.

[37] Boyle WJ, Simonet WS, Lacey DL (2003). Osteoclast differentiation and activation. *Nature*.

[38] Hsu H, Lacey DL, Dunstan CR (1999). Tumor necrosis factor receptor family member RANK mediates osteoclast differentiation and activation induced by osteoprotegerin ligand. *Proceedings of the National Academy of Sciences of the United States of America*.

[39] Phan TCA, Xu J, Zheng MH (2004). Interaction between osteoblast and osteoclast: impact in bone disease. *Histology and Histopathology*.

[40] Fuller K, Wong B, Fox S, Choi Y, Chambers TJ (1998). TRANCE is necessary and sufficient for osteoblast-mediated activation of bone resorption in osteoclasts. *Journal of Experimental Medicine*.

[41] Sugatani T, Hruska KA (2013). Down-regulation of miR-21 biogenesis by estrogen action contributes to osteoclastic apoptosis. *Journal of Cellular Biochemistry*.

[42] Hassan MQ, Maeda Y, Taipaleenmaki H (2012). miR-218 directs a Wnt signaling circuit to promote differentiation of osteoblasts and osteomimicry of metastatic cancer cells. *The Journal of Biological Chemistry*.

[43] Cheng P, Chen C, He HB (2013). miR-148a regulates osteoclastogenesis by targeting V-maf musculoaponeurotic fibrosarcoma oncogene homolog B. *Journal of Bone and Mineral Research*.

[44] Troen BR (2003). Molecular mechanisms underlying osteoclast formation and activation. *Experimental Gerontology*.

[45] Hofbauer LC, Khosla S, Dunstan CR, Lacey DL, Spelsberg TC, Riggs BL (1999). Estrogen stimulates gene expression and protein production of osteoprotegerin in human osteoblastic cells. *Endocrinology*.

[46] Khosla S, Atkinson EJ, Dunstan CR, O’Fallon WM (2002). Effect of estrogen versus testosterone on circulating osteoprotegerin and other cytokine levels in normal elderly men. *Journal of Clinical Endocrinology and Metabolism*.

[47] Shevde NK, Bendixen AC, Dienger KM, Pike JW (2000). Estrogens suppress RANK ligand-induced osteoclast differentiation via a stromal cell independent mechanism involving c-Jun repression. *Proceedings of the National Academy of Sciences of the United States of America*.

[48] Srivastava S, Toraldo G, Weitzmann MN, Cenci S, Ross FP, Pacifici R (2001). Estrogen decreases osteoclast formation by down-regulating receptor activator of NF-kappa B ligand (RANKL)-induced JNK activation. *Journal of Biological Chemistry*.

[49] Srivastava S, Weitzmann MN, Cenci S, Ross FP, Adler S, Pacifici R (1999). Estrogen decreases TNF gene expression by blocking JNK activity and the resulting production of c-Jun and JunD. *The Journal of Clinical Investigation*.

[50] Zaidi M, Blair HC, Moonga BS, Abe E, Huang CL-H (2003). Osteoclastogenesis, bone resorption, and osteoclast-based therapeutics. *Journal of Bone and Mineral Research*.

[51] Ambros V, Chen X (2007). The regulation of genes and genomes by small RNAs. *Development*.

[52] Cullen BR (2004). Transcription and processing of human microRNA precursors. *Molecular Cell*.

[53] Bentwich I, Avniel A, Karov Y (2005). Identification of hundreds of conserved and nonconserved human microRNAs. *Nature Genetics*.

[54] Lee Y, Jeon K, Lee J-T, Kim S, Kim VN (2002). MicroRNA maturation: stepwise processing and subcellular localization. *The EMBO Journal*.

[55] Lagos-Quintana M, Rauhut R, Lendeckel W, Tuschl T (2001). Identification of novel genes coding for small expressed RNAs. *Science*.

[56] Mizuno Y, Tokuzawa Y, Ninomiya Y (2009). miR-210 promotes osteoblastic differentiation through inhibition of AcvR1b. *FEBS Letters*.

[57] Mizuno Y, Yagi K, Tokuzawa Y (2008). miR-125b inhibits osteoblastic differentiation by down-regulation of cell proliferation. *Biochemical and Biophysical Research Communications*.

[58] Luzi E, Marini F, Sala SC, Tognarini I, Galli G, Brandi ML (2008). Osteogenic differentiation of human adipose tissue-derived stem cells is modulated by the miR-26a targeting of the SMAD1 transcription factor. *Journal of Bone and Mineral Research*.

[59] Li Z, Hassan MQ, Volinia S (2008). A microRNA signature for a BMP2-induced osteoblast lineage commitment program. *Proceedings of the National Academy of Sciences of the United States of America*.

[60] Huang J, Zhao L, Xing L, Chen D (2010). MicroRNA-204 regulates Runx2 protein expression and mesenchymal progenitor cell differentiation. *Stem Cells*.

[61] Kapinas K, Kessler CB, Delany AM (2009). miR-29 suppression of osteonectin in osteoblasts: regulation during differentiation and by canonical Wnt signaling. *Journal of Cellular Biochemistry*.

[62] Itoh T, Nozawa Y, Akao Y (2009). MicroRNA-141 and -200a are involved in bone morphogenetic protein-2-induced mouse pre-osteoblast differentiation by targeting distal-less homeobox 5. *Journal of Biological Chemistry*.

[63] Li H, Xie H, Liu W (2009). A novel microRNA targeting HDAC5 regulates osteoblast differentiation in mice and contributes to primary osteoporosis in humans. *The Journal of Clinical Investigation*.

[64] Hu R, Liu W, Li H (2011). A Runx2/miR-3960/miR-2861 regulatory feedback loop during mouse osteoblast differentiation. *Journal of Biological Chemistry*.

[65] Mori M, Nakagami H, Rodriguez-Araujo G, Nimura K, Kaneda Y (2012). Essential role for miR-196a in brown adipogenesis of white fat progenitor cells. *PLoS Biology*.

[66] Tomé M, López-Romero P, Albo C (2011). miR-335 orchestrates cell proliferation, migration and differentiation in human mesenchymal stem cells. *Cell Death and Differentiation*.

[67] Zhang J, Tu Q, Bonewald LF (2011). Effects of miR-335-5p in modulating osteogenic differentiation by specifically downregulating Wnt antagonist DKK1. *Journal of Bone and Mineral Research*.

[68] Schaap-Oziemlak AM, Raymakers RA, Bergevoet SM (2010). MicroRNA hsa-miR-135b regulates mineralization in osteogenic differentiation of human unrestricted somatic stem cells. *Stem Cells and Development*.

[69] Li Z, Hassan MQ, Jafferji M (2009). Biological functions of miR-29b contribute to positive regulation of osteoblast differentiation. *Journal of Biological Chemistry*.

[70] Eskildsen T, Taipaleenmäki H, Stenvang J (2011). MicroRNA-138 regulates osteogenic differentiation of human stromal (mesenchymal) stem cells in vivo. *Proceedings of the National Academy of Sciences of the United States of America*.

[71] Zhang J-F, Fu W-M, He M-L (2011). MiRNA-20a promotes osteogenic differentiation of human mesenchymal stem cells by co-regulating BMP signaling. *RNA Biology*.

[72] Zhang J-F, Fu W-M, He M-L (2011). MiR-637 maintains the balance between adipocytes and osteoblasts by directly targeting Osterix. *Molecular Biology of the Cell*.

[73] Laine SK, Alm JJ, Virtanen SP, Aro HT, Laitala-Leinonen TK (2012). MicroRNAs miR-96, miR-124, and miR-199a regulate gene expression in human bone marrow-derived mesenchymal stem cells. *Journal of Cellular Biochemistry*.

[74] Suomi S, Taipaleenmäki H, Seppänen A (2008). MicroRNAs regulate osteogenesis and chondrogenesis of mouse bone marrow stromal cells. *Gene Regulation and Systems Biology*.

[75] Sugatani T, Vacher J, Hruska KA (2011). A microRNA expression signature of osteoclastogenesis. *Blood*.

[76] Zhu LY, Qiu C, Lv JX, Xu JQ (2013). HIV-1 infection changes miRNA expression profile in the whole blood. *Bing Du Xue Bao*.

[77] Raaijmakers MHGP, Mukherjee S, Guo S (2010). Bone progenitor dysfunction induces myelodysplasia and secondary leukaemia. *Nature*.

[78] Sugatani T, Hruska KA (2009). Impaired micro-RNA pathways diminish osteoclast differentiation and function. *Journal of Biological Chemistry*.

[79] Sugatani T, Hruska KA (2007). MicroRNA-223 is a key factor in osteoclast differentiation. *Journal of Cellular Biochemistry*.

[80] Wang Y, Li L, Moore BT (2012). Mir-133a in human circulating monocytes: a potential biomarker associated with postmenopausal osteoporosis. *PLoS ONE*.

[81] Yin MT, Zhang CA, McMahon DJ (2012). Higher rates of bone loss in postmenopausal HIV-infected women: a longitudinal study. *Journal of Clinical Endocrinology and Metabolism*.

[82] Cazanave C, Dupon M, Lavignolle-Aurillac V (2008). Reduced bone mineral density in HIV-infected patients: prevalence and associated factors. *AIDS*.

[83] Yin M, Dobkin J, Brudney K (2005). Bone mass and mineral metabolism in HIV^+^ postmenopausal women. *Osteoporosis International*.

[84] Jones S, Restrepo D, Kasowitz A (2008). Risk factors for decreased bone density and effects of HIV on bone in the elderly. *Osteoporosis International*.

[85] Carr A, Miller J, Eisman JA, Cooper DA (2001). Osteopenia in HIV-infected men: association with asymptomatic lactic acidemia and lower weight pre-antiretroviral therapy. *AIDS*.

[86] Amiel C, Ostertag A, Slama L (2004). BMD is reduced in HIV-infected men irrespective of treatment. *Journal of Bone and Mineral Research*.

[87] Mondy K, Yarasheski K, Powderly WG (2003). Longitudinal evolution of bone mineral density and bone markers in human immunodeficiency virus-infected individuals. *Clinical Infectious Diseases*.

[88] Gibellini D, de Crignis E, Ponti C (2008). HIV-1 triggers apoptosis in primary osteoblasts and HOBIT cells through TNF*α* activation. *Journal of Medical Virology*.

[89] Amorosa V, Tebas P (2006). Bone disease and HIV infection. *Clinical Infectious Diseases*.

[90] Pan G, Kilby M, McDonald JM (2006). Modulation of osteoclastogenesis induced by nucleoside reverse transcriptase inhibitors. *AIDS Research and Human Retroviruses*.

[91] Gibellini D, Borderi M, de Crignis E (2007). RANKL/OPG/TRAIL plasma levels and bone mass loss evaluation in antiretroviral naive HIV-1-positive men. *Journal of Medical Virology*.

[92] Wei S, Kitaura H, Zhou P, Patrick Ross F, Teitelbaum SL (2005). IL-1 mediates TNF-induced osteoclastogenesis. *The Journal of Clinical Investigation*.

[93] Fakruddin JM, Laurence J (2003). HIV envelope gp120-mediated regulation of osteoclastogenesis via receptor activator of nuclear factor kappa B ligand (RANKL) secretion and its modulation by certain HIV protease inhibitors through interferon-gamma/RANKL cross-talk. *Journal of Biological Chemistry*.

[94] Fakruddin JM, Laurence J (2005). HIV-1 Vpr enhances production of receptor of activated NF-*κ*B ligand (RANKL) via potentiation of glucocorticoid receptor activity. *Archives of Virology*.

[95] Herbeuval J-P, Boasso A, Grivel J-C (2005). TNF-related apoptosis-inducing ligand (TRAIL) in HIV-1-infected patients and its in vitro production by antigen-presenting cells. *Blood*.

[96] Yang Y, Tikhonov I, Ruckwardt TJ (2003). Monocytes treated with human immunodeficiency virus Tat kill uninfected CD4^+^ cells by a tumor necrosis factor-related apoptosis-induced ligand-mediated mechanism. *Journal of Virology*.

[97] Wong BR, Besser D, Kim N (1999). TRANCE, a TNF family member, activates Akt/PKB through a signaling complex involving TRAF6 and c-Src. *Molecular Cell*.

[98] Pan G, Yang Z, Ballinger SW, McDonald JM (2006). Pathogenesis of osteopenia/osteoporosis induced by highly active anti-retroviral therapy for AIDS. *Annals of the New York Academy of Sciences*.

[99] Salmena L, Poliseno L, Tay Y, Kats L, Pandolfi PP (2011). A ceRNA hypothesis: the rosetta stone of a hidden RNA language?. *Cell*.

[100] Chang ST, Sova P, Peng X (2011). Next-generation sequencing reveals HIV-1-mediated suppression of T cell activation and RNA processing and regulation of noncoding RNA expression in a CD4^+^ T cell line. *mBio*.

[101] Navare AT, Sova P, Purdy DE (2012). Quantitative proteomic analysis of HIV-1 infected CD4^+^ T cells reveals an early host response in important biological pathways: protein synthesis, cell proliferation, and T-cell activation. *Virology*.

[102] Yeung ML, Bennasser Y, Myers TG, Jiang G, Benkirane M, Jeang K-T (2005). Changes in microRNA expression profiles in HIV-1-transfected human cells. *Retrovirology*.

[103] Houzet L, Yeung ML, de Lame V, Desai D, Smith SM, Jeang K-T (2008). MicroRNA profile changes in human immunodeficiency virus type 1 (HIV-1) seropositive individuals. *Retrovirology*.

[104] Chang ST, Thomas MJ, Sova P, Green RR, Palermo RE, Katze MG (2013). Next-generation sequencing of small RNAs from HIV-infected cells identifies phased microrna expression patterns and candidate novel microRNAs differentially expressed upon infection. *mBio*.

